# Type 2 Diabetes Mellitus May Exacerbate Gray Matter Atrophy in Patients With Early-Onset Mild Cognitive Impairment

**DOI:** 10.3389/fnins.2020.00856

**Published:** 2020-08-11

**Authors:** Chang Li, Zhiwei Zuo, Daihong Liu, Rui Jiang, Yang Li, Haitao Li, Xuntao Yin, Yuqi Lai, Jian Wang, Kunlin Xiong

**Affiliations:** ^1^Department of Radiology, Daping Hospital, Army Medical University, Chongqing, China; ^2^Department of Radiology, Southwest Hospital, Army Medical University, Chongqing, China; ^3^Department of Radiology, General Hospital of Western Theater Command, Chengdu, China; ^4^Department of Medical Imaging, Guizhou Provincial People’s Hospital, Guizhou, China; ^5^School of Foreign Languages and Cultures, Chongqing University, Chongqing, China

**Keywords:** type 2 diabetes mellitus, mild cognitive impairment, gray matter, atrophy, structural covariance

## Abstract

**Background:**

The precise physiopathological association between the courses of neurodegeneration and cognitive decline in type 2 diabetes mellitus (T2DM) remains unclear. This study sought to comprehensively investigate the distribution characteristics of gray matter atrophy in middle-aged T2DM patients with newly diagnosed mild cognitive impairment (MCI).

**Methods:**

Four groups, including 28 patients with early-onset MCI, 28 patients with T2DM, 28 T2DM patients with early-onset MCI (T2DM-MCI), and 28 age-, sex-, and education-matched healthy controls underwent three-dimensional high-resolution structural magnetic resonance imaging. Cortical and subcortical gray matter volumes were calculated, and a structural covariance method was used to evaluate the morphological relationships within the default mode network (DMN).

**Results:**

Overlapped and unique cortical/subcortical gray matter atrophy was found in patients with MCI, T2DM and T2DM-MCI in our study, and patients with T2DM-MCI showed lower volumes in several areas than patients with MCI or T2DM. Volume loss in subcortical areas (including the thalamus, putamen, and hippocampus), but not in cortical areas, was related to cognitive impairment in patients with MCI and T2DM-MCI. No associations between biochemical measurements and volumetric reductions were found. Furthermore, patients with MCI and those with T2DM-MCI showed disrupted structural connectivity within the DMN.

**Conclusion:**

These findings provide further evidence that T2DM may exacerbate atrophy of specific gray matter regions, which may be primarily associated with MCI. Impairments in gray matter volume related to T2DM or MCI are independent of cardiovascular risk factors, and subcortical atrophy may play a more pivotal role in cognitive impairment than cortical alterations in patients with MCI and T2DM-MCI. The enhanced structural connectivity within the DMN in patients with T2DM-MCI may suggest a compensatory mechanism for the chronic neurodegeneration.

## Introduction

Type 2 diabetes mellitus (T2DM) is a chronic metabolic disorder characterized by sustained hyperglycemia and insulin resistance correlated with multiple macrovascular and microvascular complications, which are important vascular risk factors for accelerated cognitive impairment and dementia (Allen et al., 2004). Dementia is an irreversible syndrome affecting various cognitive functions, which have been implicated in disruptions in daily function, and the primary cause of dementia worldwide is Alzheimer’s disease (AD) (Alkasir et al., 2017). While mild cognitive impairment (MCI) is a prodromal stage of dementia, patients with MCI show anosognosia and deficits in memory (Galeone et al., 2011), executive, language and visuospatial functions (Lei et al., 2016) that are not severe enough to affect a patient’s intellectual functioning and activities of daily life. Therefore, early detection and diagnosis of T2DM patients with MCI (T2DM-MCI), at least 47% of whom are likely to develop dementia (Lu et al., 2009), are crucial for timely prevention and treatment.

The clinical presentation and psychological examinations are currently the only methods used to make a definitive diagnosis of MCI because there is a lack of reliable and sensitive biomarkers for discrimination. Neurophysiological alterations have been well documented in patients with diabetes (Chen et al., 2012; Hughes et al., 2013; Moran et al., 2013; Garcia-Casares et al., 2014; Mehta et al., 2014; Wu et al., 2017) or MCI (Pennanen et al., 2004; Tondelli et al., 2012; Zheng et al., 2014; Zeifman et al., 2015; Lei et al., 2016; Gong et al., 2017) and have the potential to become a preclinical hallmark of macrostructural changes that provide supplementary information for detecting the condition early, monitoring the progression and evaluating interventions. Volumetric reductions in gray matter in the frontal, temporal, hippocampal, and occipitoparietal regions have been demonstrated in patients with diabetes (Chen et al., 2012; Hughes et al., 2013; Moran et al., 2013; Garcia-Casares et al., 2014; Mehta et al., 2014; Wu et al., 2017). Meanwhile, neuroanatomical changes in the gray matter of patients with MCI were predominantly located in medial temporal areas, such as the hippocampus and entorhinal cortex (Pennanen et al., 2004; Zeifman et al., 2015; Gong et al., 2017), and in the frontal, parietal and occipital cortices (Tondelli et al., 2012; Zheng et al., 2014; Lei et al., 2016). Some of these structural alterations in patients with diabetes or MCI appeared to overlap; however, many of the above-mentioned neuropathological studies of T2DM excluded patients with dementia but did not rule out patients with concomitant MCI (Chen et al., 2012; Moran et al., 2013; Garcia-Casares et al., 2014; Mehta et al., 2014); the inclusion of these subjects in statistical analyses may reflect the results of combining patients with T2DM with normal cognitive function and those with T2DM-MCI. Therefore, it was unclear that those overlapped changes were related to T2DM, MCI or the combined effects of T2DM and MCI. Actually, T2DM patients with MCI may have a lower total gray matter volume than T2DM patients with normal cognition (Groeneveld et al., 2018). Therefore, whether the structural alterations in patients with T2DM-MCI were a consequence of diabetes or the presence of accelerated cognitive impairment warrants further examination.

Most previous studies utilized voxel-based morphometry (VBM) to calculate volumetric changes in gray matter in patients with T2DM (Chen et al., 2012; Moran et al., 2013; Garcia-Casares et al., 2014; Wu et al., 2017) and MCI (Tondelli et al., 2012; Zheng et al., 2014; Zeifman et al., 2015; Lei et al., 2016). However, heavy image smoothing with a Gaussian filter kernel and cortical folding in VBM processing may cause bias in detecting volumetric changes, especially for the small cerebral regions. Compared with VBM, the surface-based method can provide a more accurate measure of gray matter volume in a subvoxel scale (Greve et al., 2013) and was more sensitive in measuring aged-related reductions in gray matter in healthy aging (Hutton et al., 2009). In addition, very few structural studies on T2DM or MCI have been conducted in middle-aged subjects, which may have relatively fewer cognitive complaints and are less influenced by normal aging. With fewer confounding factors, the accuracy of the evaluation of early anatomical changes that may indicate a progressive process could be improved. Furthermore, studies of gray matter changes in T2DM-MCI are rare and mainly focus on the cortical variations (Zhang et al., 2014; Groeneveld et al., 2018; Li et al., 2018), while subcortical and hippocampal candidates affecting cognitive impairment and possible structural connectivity alterations in patients with T2DM-MCI, which could represent the specific effects of T2DM on MCI, have not been well-defined.

In light of these previous studies, we attempted to achieve a comprehensive evaluation of the gray matter morphological changes and characteristics of their distribution in middle-aged patients with T2DM-MCI by conducting comparisons with patients with MCI, patients with T2DM and cognitively normal subjects using a surface-based technique. We hypothesized that the MCI, T2DM and T2DM-MCI groups would have different anatomical patterns of gray matter alterations, and different anatomical patterns of gray matter alterations would be correlated with neuropsychological impairments. Our findings regarding determination of the structural connectivity model, etiology and location of morphological alterations in patients with T2DM-MCI may help identify the best opportunity to implement a preventive therapeutic program.

## Materials and Methods

### Subjects

Thirty-one patients with T2DM-MCI, 29 patients with MCI and 28 patients with T2DM were recruited from the outpatient clinic of our hospital. The diagnosis of T2DM was made according to the 1999 criteria proposed by the World Health Organization (Alberti and Zimmet, 1998). MCI was defined based on the diagnostic criteria established in the 2006 European Alzheimer’s Disease Consortium (Portet et al., 2006) and included memory complaints, Montreal Cognitive Assessment (MoCA) score < 26, Mini-Mental State Examination (MMSE) score > 24, and normal activities of daily living (ADL) score. The exclusion criteria included a history of stroke, brain injury, alcohol use disorder, epilepsy, Parkinson’s disease, major depression (a total 24-item Hamilton Depression Rating Scale [HAM-D] score > 20), AD (met diagnostic criteria based on Diagnostic and Statistical Manual of Mental Disorders, Fourth Edition [DSM-IV] and MMSE score < 24), other psychiatric or neurological disorders or any contraindications for MRI. Ultimately, 28 patients with T2DM-MCI (17 females), 28 patients with MCI (20 females) and 28 patients with T2DM (18 females) were enrolled in our study. Twenty-eight age-, sex-, and education-matched healthy controls (HCs) (15 females) who had no history of an alcohol or substance use disorder, vascular risk factors, psychiatric disease, traumatic brain injury, and neurological disease were recruited from the general public through advertisements.

All participants were right-handed and were 40 to 65 years old (to reduce the impact of normal aging), and patients with MCI or T2DM-MCI were newly diagnosed with MCI. All participants included in the study signed an informed consent form before the study started. This study was carried out in accordance with the Declaration of Helsinki. The protocol was approved by the Medical Ethics Committee of our hospital.

### Clinical, Biochemical, and Neuropsychological Assessments

The height, weight, body mass index (BMI) and blood pressure of each participant were measured using a standardized protocol.

Blood samples were collected in the morning after fasting overnight. A blood biochemical analyzer (AU2700; Olympus, Japan) was used to enzymatically measure fasting plasma glucose (FPG), glycosylated hemoglobin (HbA_1C_), total cholesterol (TC), triglyceride (TG), high-density lipoprotein cholesterol (HDL), and low-density lipoprotein cholesterol (LDL), which are known cardiovascular risk factors for cognitive impairment (McCrimmon et al., 2012).

All participants participated in interviews and received independent neuropsychological examinations, including the MoCA, MMSE, ADL, HAM-D, and Trail-Making Test (TMT).

### MRI Acquisition

A Siemens 3.0-Tesla Trio Tim MRI scanner (Siemens AG, Erlangen, Germany) equipped with a 12-channel phase-array head coil was used to acquire three-dimensional high-resolution structural images. The subjects were placed in the supine position and were asked to keep their head as still as possible during image acquisition. Before the scanning of structural images, T1-weighted, T2-weighted and fluid-attenuated inversion recovery (FLAIR) MRI sequences were implemented for each subject to exclude organic diseases and white matter hyperintensities. The following magnetization-prepared rapid gradient echo (MPRAGE) acquisition parameters were used: repetition time (TR) = 1900 ms; echo time (TE) = 2.52 ms; inversion time (TI) = 1100 ms; flip angle = 9°; field of view (FOV) = 256 mm × 256 mm; slice thickness = 1 mm; number of slices = 176; and voxel size = 1 mm × 1 mm × 1 mm.

### MRI Analysis

We first verified that all images were not impacted by head movement. Volume segmentation and cortical surface reconstruction of structural images were carried out using FreeSurfer software (version 5.3.0, Massachusetts General Hospital, Boston, MA, United States^1^). The post-processing stream has been described in detail in previous studies (Dale et al., 1999; Fischl and Dale, 2000). After the automated processing, the reconstructed cortical surfaces and subcortical segmentation were investigated to determine whether they followed gray matter boundaries and intensity borders, respectively, and if they were not aligned, each error was manually modified for the proper segmentation. The volumes of subcortical areas, including the thalamus, caudate, putamen, pallidum, hippocampus, amygdala, and nucleus accumbens, were extracted from the reconstructions. In addition, we further segmented the hippocampus to observe the role of structural variations in hippocampal subfields. The segmentations of the hippocampus include the hippocampal tail (HT), subiculum, cornu ammonis (CA) 1, hippocampal fissure (HF), presubiculum, parasubiculum, molecular layer (ML), granule cell layer of dentate gyrus (DG), CA3, CA4, fimbria, and hippocampus-amygdala-transition-area (HATA) (Iglesias et al., 2015). According to the study of Andrews-Hanna et al. (2010), we used the left posterior cingulate cortex (PCC), which is the critical hub of the default mode network (DMN) (Andrews-Hanna et al., 2010; Dunn et al., 2014; Cui et al., 2015; Chirles et al., 2017), as the seed region to calculate its structural connectivity based on gray matter volume with 10 other key subsections of the DMN, including the bilateral medial prefrontal cortex, precuneus, temporal pole, lateral temporal cortex and hippocampus. The standard regions of interest (ROIs) were defined based on the Automated Anatomical Labeling (AAL) template (Tzourio-Mazoyer et al., 2002) and Desikan-Killiany parcellation (Desikan et al., 2006).

### Statistical Analysis

Statistical analyses were performed using SPSS 22.0 software (IBM, Inc., Armonk, NY, United States). Comparisons of demographic features, standard clinical laboratory testing measurements and neuropsychological scores among the four groups were performed using the χ^2^ test, independent two-sample *t*-test, one-way analysis of variance (ANOVA) or Kruskal–Wallis one-way ANOVA. Differences in the cortical volume among the four groups were evaluated using the general linear model (GLM). A whole-brain statistical threshold correction was performed using the Monte Carlo simulation method (Hagler et al., 2006), and statistical significance was set at a clusterwise-corrected *p*-value < 0.05. To assess the differences in volumes of subcortical areas and hippocampal subfields among the four groups, one-way ANOVA followed by the least-squares difference (LSD) *post hoc* test or Kruskal–Wallis one-way ANOVA followed by all pairwise corrections was performed. Pearson correlation analysis was used to determine the structural covariance based on gray matter volume. To assess the differences in correlation coefficients between groups, Snedecor’s method (Snedecor and William, 1989) was used to transform *r* values to *z* values. Where appropriate, the Bonferroni correction was applied to correct for multiple comparisons that involved multiple brain areas, and Bonferroni-corrected *p*-values < 0.05 were considered significant. Correlations between the altered biochemical measurements/neuropsychological scores and gray matter volumes were analyzed using partial Pearson correlation analysis or partial Spearman correlation analysis with adjustments for age, sex, and education level. The *p* level indicating statistical significance was set at < 0.01 without correction for multiple comparisons to assess potential trends.

## Results

### Participant Characteristics

Demographic information, standard laboratory testing measurements and neuropsychological data by group are presented in Table 1. No significant differences in age, sex, education level, duration of T2DM, SBP, DBP, TC level, or TG level were detected between groups (*p* > 0.05). There were significant differences in the BMI, FPG, HbA_1c_, HDL levels and LDL levels, the time to complete the TMT-A and TMT-B, and the MoCA and MMSE scores between the four groups (*p* < 0.05). *Post hoc* comparisons showed that both the HCs and patients with MCI had significantly lower FPG and HbA_1c_ levels and greater HDL levels than patients with T2DM-MCI and patients with T2DM; both patients with MCI and patients with T2DM-MCI had significantly greater TMT-A completion times and lower MoCA scores than HCs and patients with T2DM; patients with MCI had significantly greater TMT-B completion times and lower MMSE scores than HCs and patients with T2DM.

**TABLE 1:** Demographic features, biochemical measurements, and neuropsychological performance.

Characteristic	HC (*n* = 28)	MCI (*n* = 28)	T2DM-MCI (*n* = 28)	T2DM (*n* = 28)	Diagnosis effect	*p*-value
Age, years	53.9 ± 1.2	56.6 ± 1.1	56.5 ± 1.2	54.8 ± 1.2	*H* = 4.32	0.229^a^
Sex, female : male	15:13	20:8	17:11	18:10	χ^2^ = 1.98	0.576^b^
Education, years	12.3 ± 0.7	11.3 ± 0.6	11.0 ± 0.5	12.4 ± 0.6	*H* = 3.13	0.372^a^
Duration of T2DM	-	-	8.2 ± 1.0	8.3 ± 1.3	*t* = 0.04	0.965^c^
BMI, kg/m^2^	23.5 ± 0.5	23.1 ± 0.4	24.8 ± 0.5	25.8 ± 1.2	*H* = 8.59	0.035^a^*
SBP, mm Hg	127.4 ± 3.8	128.8 ± 3.2	131.4 ± 3.3	127.1 ± 2.8	*H* = 1.65	0.648^a^
DBP, mm Hg	79.0 ± 1.7	78.4 ± 1.9	80.1 ± 1.9	78.8 ± 1.9	*F* = 0.16	0.921^d^
FPG, mmol/L	5.5 ± 0.1	5.5 ± 0.1	10.4 ± 0.6	8.6 ± 0.6	*H* = 73.05	< 0.0001^d^ *
HbA_1C_ (%)	5.5 ± 0.1	5.6 ± 0.1	9.8 ± 0.6	8.8 ± 0.3	*H* = 83.21	< 0.0001^a *^
Total cholesterol, mmol/L	5.1 ± 0.2	5.4 ± 0.1	5.3 ± 0.2	4.8 ± 0.3	*H* = 7.34	0.062^a^
TG, mmol/L	1.5 ± 0.1	1.9 ± 0.5	2.4 ± 0.4	2.6 ± 0.7	*H* = 5.54	0.137^a^
HDL cholesterol, mmol/L	1.4 ± 0.1	1.5 ± 0.1	1.2 ± 0.1	1.1 ± 0.0	*F* = 9.33	< 0.0001^d *^
LDL cholesterol, mmol/L	3.1 ± 0.1	3.4 ± 0.1	3.4 ± 0.2	2.9 ± 0.1	*F* = 3.51	0.018^d*^
TMT-A	48.1 ± 2.4	60.1 ± 4.2	64.6 ± 4.2	49.6 ± 4.0	*F* = 4.54	0.005^d*^
TMT-B	60.1 ± 4.0	79.3 ± 4.9	99.8 ± 8.7	68.8 ± 5.8	*H* = 19.58	< 0.0001^a*^
MoCA score	27.7 ± 0.2	22.0 ± 0.5	22.1 ± 0.4	27.1 ± 0.2	*H* = 84.93	< 0.0001^a *^
MMSE score	28.5 ± 0.2	27.1 ± 0.3	27.8 ± 0.2	28.3 ± 0.2	*H* = 13.97	0.003^a *^

*HC, healthy control; MCI, mild cognitive impairment; T2DM, type 2 diabetes mellitus; BMI, body mass index; SBP, systolic blood pressure; DBP, diastolic blood pressure; FPG, fasting plasma glucose; HbA_1C_, glycosylated hemoglobin; TG, triglyceride; HDL, high density lipoprotein; LDL, low density lipoprotein; Trail-Making Test, TMT; MoCA, Montreal Cognitive Assessment; MMSE, mini mental state exam. Continuous variables are expressed as the means ± SEM. ^∗^Significant p-values < 0.05. ^a^Indicates p-values for the Kruskal–Wallis one-way ANOVA. ^b^Indicates p-values for the chi-square test. ^c^Indicates p-values for the independent two-sample t-test. ^d^Indicates p-values for one-way ANOVA.*

### Cortical Volume

**FIGURE 1:**
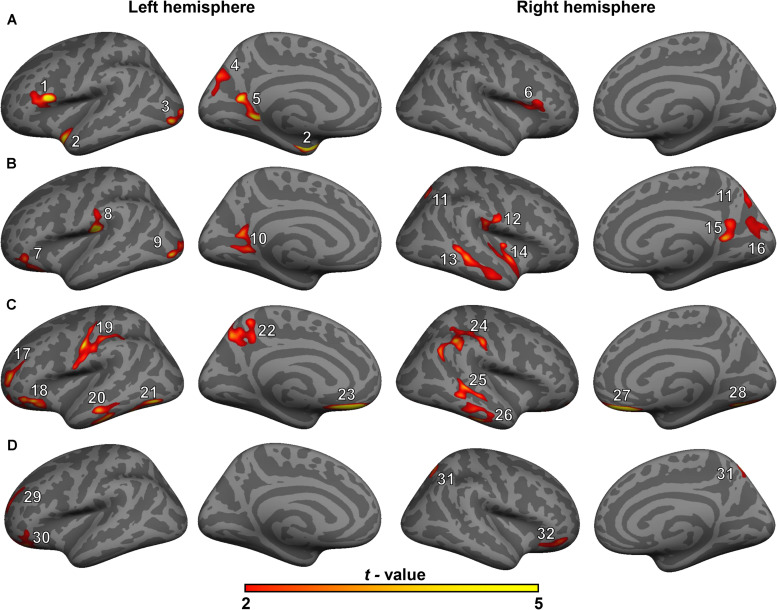
Surface maps of significant differences in cortical volumes between healthy controls (HCs) and patients with mild cognitive impairment (MCI) **(A)**, between HCs and patients with type 2 diabetes mellitus and MCI (T2DM-MCI) **(B)**, between patients with MCI and patients with T2DM-MCI **(C)**, and between patients with T2DM and patients with T2DM-MCI **(D)**. Differences in cortical volume are presented on inflated cortical surfaces (clusterwise-corrected *p*-value < 0.05). Dark gray indicates gyri; light gray indicates sulci. The color bar represents *t*-values ranging from 2 to 5 (red to yellow). The numerals refer to the cluster numbers listed in Table 2.

The GLM analysis results of cortical volume are displayed in Figure 1 and Table 2. Compared with the HC group, MCI group or T2DM group, the T2DM-MCI group showed smaller volumes in 10, 12, or 4 cortical clusters, respectively. Compared with the HC group, the MCI group had smaller cortical areas mainly located in the left hemisphere. However, no significant difference in cortical volume was found between the HC and T2DM groups.

**TABLE 2:** Surface-based cluster summary of significant cortical changes.

Cluster number	*t*-Value Maximum	Size (mm^2^)	MNI coordinates of peak vertex			CWP	Anatomical location
			*X*	*Y*	*Z*		
*HC* vs. *MCI*							
1	6.374	882.25	−54.0	17.2	17.7	0.0002	L pars opercularis
2	5.653	624.73	−25.5	−1.2	−29.5	0.0042	L entorhinal cortex
3	4.741	681.83	−39.1	−86.9	−8.8	0.0018	L lateral occipital cortex
4	3.663	571.54	−17.6	−79.4	31.4	0.0078	L superior parietal cortex
5	5.413	561.52	−11.0	−58.3	12.8	0.0086	L precuneus
6	3.714	591.94	44.8	6.7	7.5	0.0070	R precentral cortex
*HC* vs. *T2DM-MCI*							
7	3.843	613.67	−43.2	37.7	−14.0	0.0048	L pars orbitalis
8	8.061	562.66	−43.9	−22.4	21.1	0.0086	L supramarginal gyrus
9	4.728	732.01	−39.2	−86.9	−7.5	0.0010	L lateral occipital cortex
10	4.104	687.45	−10.1	−58.1	11.4	0.0016	L precuneus
11	3.588	510.02	20.8	−63.1	45.2	0.0195	R superior parietal cortex
12	3.758	523.84	53.8	−21.4	19.5	0.0167	R supramarginal gyrus
13	4.241	1023.73	58.3	−34.3	−8.6	0.0002	R middle temporal cortex
14	4.093	846.49	41.0	−13.7	−12.0	0.0004	R superior temporal cortex
15	4.493	458.40	14.9	−53.9	8.6	0.0402	R isthmus cingulate cortex
16	2.694	722.45	5.3	−81.9	21.9	0.0014	R cuneus
*MCI* vs. *T2DM-MCI*							
17	4.634	485.16	−33.2	48.8	8.7	0.0235	L rostral middle frontal cortex
18	4.751	922.47	−42.1	27.4	−14.3	0.0002	L lateral orbitofrontal cortex
19	5.112	1364.60	−45.8	−13.7	27.9	0.0002	L post-central cortex
20	4.467	722.46	−54.2	−32.1	−25.8	0.0012	L inferior temporal cortex
21	5.728	482.85	−41.8	−45.5	−15.0	0.0247	L fusiform cortex
22	4.206	979.14	−6.1	−68.6	43.7	0.0002	L precuneus
23	10.659	1088.50	−15.0	31.6	−22.0	0.0002	L lateral orbitofrontal cortex
24	4.705	1463.05	48.1	−44.4	37.2	0.0002	R supramarginal gyrus
25	4.374	502.35	44.4	−37.9	−1.0	0.0213	R banks of the superior temporal sulcus
26	4.861	718.75	58.2	−26.7	−27.9	0.0014	R inferior temporal cortex
27	7.946	1157.67	5.3	32.8	−24.0	0.0002	R medial orbitofrontal cortex
28	5.721	564.98	29.4	−68.2	−8.8	0.0108	R fusiform cortex
*T2DM* vs. *T2DM-MCI*							
29	4.243	687.61	−24.7	37.3	−9.3	0.0016	L lateral orbitofrontal cortex
30	3.240	487.47	−22.5	54.5	18.3	0.0225	L rostral middle frontal cortex
31	4.340	479.08	12.2	−68.9	54.9	0.0306	R superior parietal cortex
32	3.663	483.89	37.0	40.5	−9.1	0.0284	R pars orbitalis

*HC, healthy control; MCI, mild cognitive impairment; T2DM, type 2 diabetes mellitus; CWP, cluster wise p-value; MNI, Montreal Neurological Institute; L, left hemisphere; R, right hemisphere.*

### Subcortical Volume

**FIGURE 2:**
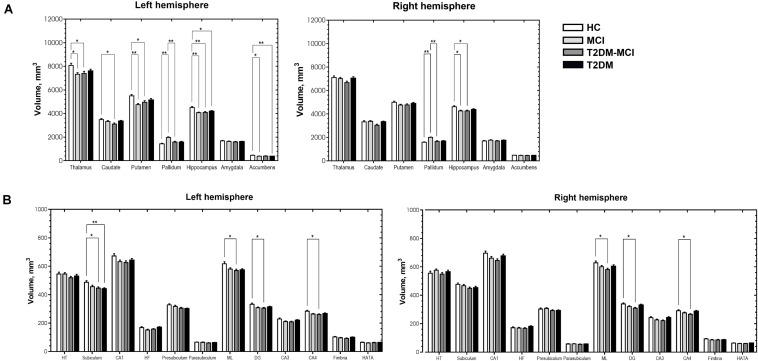
Significant differences in subcortical volume **(A)** and volume of the hippocampal subfields **(B)** among the HCs, patients with MCI, patients with type 2 diabetes mellitus and mild cognitive impairment (T2DM-MCI) and patients with type 2 diabetes mellitus (T2DM). *Bonferroni-corrected *p*-value < 0.05. **Bonferroni-corrected *p*-value < 0.001. The error bars indicate standard errors. HT, hippocampal tail; CA, cornu ammonis; HF, hippocampal fissure; ML, molecular layer; DG, granule cell layer of dentate gyrus; HATA, hippocampus-amygdala-transition-area.

Figure 2A shows subcortical volumes of the four groups (Bonferroni-corrected *p*-value < 0.05). Both the MCI and T2DM-MCI groups showed significantly lower volumes than the HC group in the left thalamus, putamen, and bilateral hippocampus. The MCI group also had significantly lower volumes in the left nucleus accumbens and greater volumes in the bilateral pallidum than the HC group, and the T2DM-MCI group also had significantly lower volumes in the left caudate than the HC group and lower volumes in the bilateral pallidum than the MCI group. The T2DM group had significantly lower volumes in the left hippocampus and nucleus accumbens than the HC group.

We further compared the volume of the hippocampal subfields (Figure 2B) and found that the T2DM-MCI group had significantly lower volumes in the bilateral ML, DG, CA4 and left subiculum than the HC group, and the T2DM group had significantly lower volumes in the left subiculum than the HC group (Bonferroni-corrected *p*-value < 0.05).

### Structural Covariance

**FIGURE 3:**
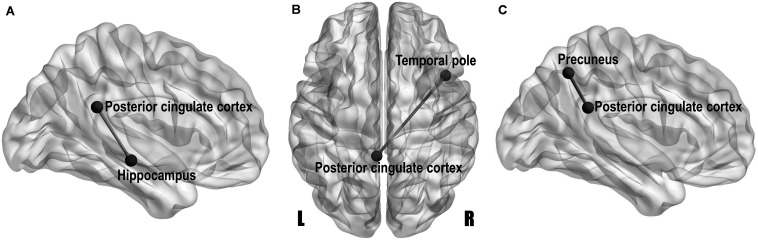
Network-style maps of significant differences in structural covariance within the default mode network between the HCs and patients with MCI **(A)**, between the HCs and patients with type 2 diabetes mellitus and MCI (T2DM-MCI) **(B)**, and between the MCI and T2DM-MCI groups **(C)**. Differences in correlation coefficients for gray matter volume are presented on a standard translucent brain. Bonferroni-corrected *p*-value < 0.05.

Volumetric covariance analysis was specifically performed for key regions involved in the DMN, and the obtained differences in correlation coefficients between groups (Bonferroni-corrected *p*-value < 0.05) are shown in the style of a brain network with the BrainNet Viewer^2^ in Figure 3. The MCI group showed a significantly lower correlation strength between the left PCC and left hippocampus than the HC group (*Z* = 2.85, Bonferroni-corrected *p* = 0.043) (Figure 3A), and the T2DM-MCI group showed a significantly lower correlation strength between the left PCC and right temporal pole than the HC group (*Z* = 3.00, Bonferroni-corrected *p* = 0.027) (Figure 3B). The T2DM-MCI group showed a significantly greater correlation strength between the left PCC and left precuneus than the MCI group (*Z* = −3.00, Bonferroni-corrected *p* = 0.028) (Figure 3C). No difference in correlation strength between the T2DM group and the other three groups was found.

### Correlation Analysis

**FIGURE 4:**
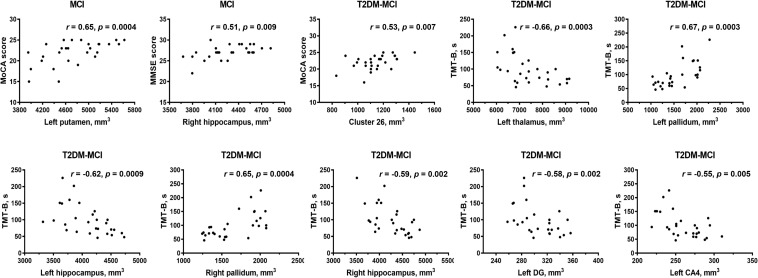
Partial correlation analysis results. CA, cornu ammonis; DG, granule cell layer of dentate gyrus; ML, molecular layer; TMT, Trail-Making Test; MoCA, Montreal Cognitive Assessment; MMSE, Mini-Mental State Examination. Significant *p*-value < 0.01.

No standard laboratory testing measurements showed significant correlation with altered gray matter volumes (*p* > 0.01) after adjusting for age, sex and education level. Gray matter volume in the left putamen and right hippocampus in patients with MCI showed positive correlations with cognitive function (measured by MMSE or MoCA) after adjusting for age, sex and education level (Figure 4). In patients with T2DM-MCI, after adjusting for age, sex and education level, volume in the cluster 26, left thalamus, and bilateral hippocampus showed negative correlations with TMT-B completion time; surprisingly, the volume of the left pallidum and right pallidum showed positive correlations with TMT-B completion time. Regarding the hippocampal subfields, volume in the left DG and left CA4 showed negative correlations with TMT-B completion time (Figure 4).

## Discussion

Gray matter atrophy of the temporal, occipital and parietal cortical regions in the patients with MCI was found in our study, which was consistent with previous studies (Pennanen et al., 2004; Tondelli et al., 2012; Zheng et al., 2014; Zeifman et al., 2015; Lei et al., 2016; Gong et al., 2017). The patients with T2DM-MCI showed a relatively extensive pattern of cortical gray matter loss involving the temporal, occipital, parietal, and cingulate cortical regions. However, inconsistent with previous structural studies in patients with diabetes (Chen et al., 2012; Hughes et al., 2013; Moran et al., 2013; Garcia-Casares et al., 2014; Mehta et al., 2014; Wu et al., 2017), the patients with T2DM in our study did not show any cortical changes compared with HCs, and except for the sample bias, a possible reason for this inconsistency may be that patients with concomitant MCI may be involved in these studies, suggesting that neuropathological studies of T2DM in the future should exclude not only patients with dementia but also patients with MCI; otherwise, the results should be explained with caution. Interestingly, if we consider the comparison results between patients with MCI and patients with T2DM-MCI as the “impacts of T2DM” on gray matter volume in patients with T2DM-MCI and the comparison results between patients with T2DM and patients with T2DM-MCI as the “impacts of MCI” on gray matter volume in patients with T2DM-MCI, it appears that the “impacts of T2DM” on cortical volume are greater than the “impacts of MCI” on cortical volume. Patients with T2DM did not exhibit cortical alterations and cardiovascular risk factors were independent of cortical changes. This contradiction may indirectly illustrate that T2DM itself may not directly impair the cortical volume but may exacerbate cortical atrophy in specific brain regions in patients with T2DM-MCI. Clusters 3 and 9 (peak vertices in the left lateral occipital cortex) and clusters 5 and 10 (peak vertices in the left precuneus) showed similarities in shape and location (Figure 1). Although the comparison between the MCI and T2DM-MCI groups did not show any significant differences in these areas, the T2DM-MCI group did have a smaller left precuneus (cluster 22) than the MCI group with a disparate shape and location relative to cluster 5/cluster 10. The precuneus is a key component in conducting visuospatial imagery, episodic memory retrieval, self-information processing and consciousness (Cavanna and Trimble, 2006). Recent functional studies have shown that metabolism (Franzmeier et al., 2017) and functional connectivity (Chirles et al., 2017) of the precuneus in patients with MCI were reduced, which could be correlated with accelerated brain atrophy (Chirles et al., 2017), as well as that the cognitive reserve of patients with MCI may benefit from protective or therapeutic interventions for sustaining or enhancing functional connectivity of the precuneus (Chirles et al., 2017). Hypoperfusion in the precuneus in T2DM was also found in a cerebral perfusion study using the arterial spin-labeling technique (Cui et al., 2017). Although that study possibly recruited patients with T2DM-MCI, hypoperfusion in the precuneus was markedly associated with increased insulin resistance in patients (Cui et al., 2017), suggesting that T2DM may play an important role in dysregulated cerebral perfusion of the precuneus. Moreover, of all the cortical alterations, only volume of the right inferior temporal cortex (cluster 26) showed a positive association with the MoCA score in patients with T2DM-MCI. The anatomical location of cluster 26 partially overlapped with that of cluster 13, and these areas were not significantly altered in the MCI or T2DM groups, which provides additional evidence that T2DM may accelerate cortical atrophy or cognitive impairment.

Independent of cortical volume, volumetric decreases in the left hippocampus and nucleus accumbens were found in patients with T2DM, suggesting that T2DM may tend to impair subcortical areas not the cortex. Gray matter atrophy of the left thalamus, putamen, and bilateral hippocampus occurred synchronously in the T2DM-MCI and MCI groups and all displayed significant relationships with cognitive impairments (assessed using the TMT, MMSE, and MoCA), suggesting that MCI may be more responsible for atrophy of these areas. Hippocampal volume loss has been proposed as a potential imaging marker for the diagnosis and prognosis of MCI (Pennanen et al., 2004; Zheng et al., 2014; Zeifman et al., 2015; Gong et al., 2017). It has been widely thought to be associated with memory decline in the course of AD (de Jong et al., 2008) and has also been found in patients with T2DM (Chen et al., 2012; Moran et al., 2013; Verdile et al., 2015). The neuropsychological assessment comparison showed no significant differences in MMSE scores between the T2DM-MCI and MCI/T2DM/HC groups, while the MCI group had lower MMSE scores than the HC and T2DM groups, which implied that the MCI group may have had more serious cognitive impairments than the T2DM-MCI group in this study. However, the subfield analysis of the hippocampus revealed that the T2DM-MCI group had significantly smaller volumes in the left subiculum and the bilateral ML, DG, and CA4; the T2DM group had significantly smaller volumes in the left subiculum; and the MCI group showed no alterations in the hippocampal subfields. Furthermore, volumes of the left DG and CA4 of the T2DM-MCI group were negatively associated with cognitive performance. Therefore, patients with T2DM-MCI, which potentially had less severe cognitive impairments, manifested more apparent gray matter losses in the hippocampal subfields, indicating that a combination of hyperglycemia, insulin resistance and other risk factors for diabetes may have exacerbated brain atrophy in specific hippocampal subfields. A surprising finding of this study was that the volume of the bilateral pallidum in the MCI group was greater than that in both the HC and T2DM-MCI groups and was positively correlated with TMT-B completion time. One possible explanation for this alteration may be a compensatory response to damaged cerebral perfusion in the pallidum (Dai et al., 2009). Similar findings were reported in previous studies (Lee et al., 2013; Yi et al., 2016), which suggested that enlargement of gray matter may have represented hypertrophy of reactive neurons and an inflammatory course in the early preclinical stage of a chronic physiopathological trajectory and that neurodegeneration of the pallidum may eventually occur. Another possible explanation may be the small sample size, which may be related to data bias. In these cortical and subcortical areas that displayed significant differences between the HC and MCI/T2DM-MCI groups, only atrophic subcortical areas showed significant associations with poorer cognitive abilities. This finding highlights the major contribution of subcortical areas, especially the thalamus, putamen, and hippocampus, to cognition in patients with MCI and T2DM-MCI and suggests that the correlation between brain atrophy and cognitive impairment may be stronger in the subcortical regions than cortical regions. In fact, damage to subcortical regions could result in worse cognitive abilities than damage to cortical regions (Turunen et al., 2013), and volumetric decreases in subcortical structures are closely associated with cognitive dysfunction, which are independent of cortical alterations, as described in studies of elderly subjects (de Jong et al., 2012), patients with AD (de Jong et al., 2008), and patients with type 1 diabetes mellitus (van Duinkerken et al., 2014). These findings may be due to the dissociable functional pattern in cortical and subcortical regions, which are prone to exhibiting correlations with distinct cognitive functions (Matias-Guiu et al., 2018). Moreover, impaired regional cerebrovascular resistance and amyloid deposition are particularly prominent in subcortical areas in patients with MCI (Nation et al., 2013), which may also contribute to the independent role of subcortical areas in cognitive impairment.

Resting-state functional MRI studies have reported significantly disrupted functional connectivity in patients with MCI or T2DM, and these changes were particularly prominent in the DMN (Dunn et al., 2014; Cui et al., 2015; Chirles et al., 2017). As another powerful brain connectivity approach, structural covariance can provide differentially highlighted connectivity characteristics and network-level features that reflect genetic effects, maturational influences and experience-related plasticity (Evans, 2013; Clos et al., 2014). However, few investigations have focused on structural covariance in MCI or T2DM. In our study, an attenuated structural relationship within the DMN was found between the left PCC and hippocampus in patients with MCI, which may be related to the gray matter atrophy in the left hippocampus identified in our study. The PCC and hippocampus are anatomically interconnected, and their functional communications are strongly correlated with episodic memory (Dunn et al., 2014). Therefore, it is quite natural that functional disconnections between the PCC and hippocampus are associated with impairments in episodic memory in patients with MCI (Dunn et al., 2014). Although there were no volumetric reductions in either the PCC or the temporal pole, the structural connectivity between the left PCC and right temporal pole was attenuated, indicating that structural covariance can identify distinct structural impairments that conventional volumetric comparisons cannot reveal. Socioemotional control in participants has been linked to the temporal pole and patients with semantic dementia accompanied by damage in the right temporal pole show alterations in personality and socially appropriate behavior (Olson et al., 2007). In addition, decreased perfusion of the PCC and temporal pole in MCI and AD has been linked to visual perceptual impairments (Alegret et al., 2010). The T2DM-MCI group showed enhanced structural connectivity between the left PCC and precuneus relative to patients with MCI, which could also be seen as the effects of diabetes on structural relationships within the DMN. The PCC and precuneus together form the strongest hub in the cortical gray matter, and this hub plays a central role in the DMN to maintain normal information communication and synthesis, which may be crucial for self-referential mental representations during rest (Dastjerdi et al., 2011). As previously mentioned, the T2DM-MCI group also showed a volumetric reduction in the left precuneus compared with the MCI group. Therefore, the increased local structural interactions between the PCC and precuneus likely indicate a compensatory response to the atrophy of the precuneus and is cognitive impairment and not just an anomalous pattern of imbalanced structural alterations.

There were several limitations of this investigation. First, this was a cross-sectional study with a relatively small sample size. Any structural alterations determined in our study should be interpreted with caution, and a greater diversity in the duration of disease should be studied longitudinally with a larger sample size in the future. Second, the various medications administered to patients with T2DM may have affected these brain structures, and it was difficult to identify whether some of the structural alterations were secondary to the potential influences of medications. Insulin, baseline FPG and other co-morbidities were not recorded and may also impact cerebral structures. In addition, the classification of MCI includes amnestic, single domain and multiple domain; however, an agreement on the identification of subtypes among patients with MCI has not been reached. Thus, the heterogeneous etiology and symptoms of MCI may have led to the diffuse results with diminished correlations between cognitive performance and specific altered regions. Third, only the MMSE, MoCA and TMT were used to evaluate the cognitive function of subjects, and more detailed and specialized neuropsychological assessments may be useful for obtaining more robust results. The strengths of this study are that, to the best of our knowledge, this study is the first to examine structural alterations in subcortical regions and hippocampal subfields and the changed structural connectivity patterns in the DMN in a cohort of well-phenotyped T2DM patients with early-onset MCI.

## Conclusion

The findings of the present study strongly suggest that reductions in gray matter volume related to T2DM or MCI are independent of cardiovascular risk factors. Furthermore, T2DM may exacerbate cortical and subcortical atrophies in specific brain regions, which may be mainly associated with MCI. The dissociable pattern of cortical and subcortical alterations with cognitive impairment stresses the crucial contribution of subcortical areas in cognitive decline in patients with MCI and T2DM-MCI. Moreover, structural covariance can be successfully used to supplement structural studies in MCI and T2DM-MCI through the calculation of the structural relationships within the cerebral areas.

## Data Availability Statement

The datasets generated for this study are available on request to the corresponding author.

## Ethics Statement

The studies involving human participants were reviewed and approved by Medical Ethics Committee of Southwest Hospital. The patients/participants provided their written informed consent to participate in this study.

## Author Contributions

JW, KX, and HL designed the experiments. CL, DL, ZZ, YLi, and XY carried out of the experiments. ZZ, CL, and RJ analyzed the imaging results. ZZ, CL, and YLa wrote the manuscript. All authors contributed to manuscript revision, read and approved the submitted version.

## Conflict of Interest

The authors declare that the research was conducted in the absence of any commercial or financial relationships that could be construed as a potential conflict of interest.

## Footnotes

1 http://surfer.nmr.mgh.harvard.edu

2 http://www.nitrc.org/projects/bnv/
